# Dr. Knight, on the Grinder's Asthma

**Published:** 1831-01-01

**Authors:** 


XXXIV.

ON THE GRINDERS' ASTHMA. By ARNOLD KNIGHT, M.D. Physician to the Sheffield Infirmary.

[North of England Medical and Surgical Journal, No. I.]

ANOTHER medical journal has commenced its career, with modest pretensions, but with very useful designs—"influenced only by a sincere desire of assisting the dissemination of knowledge." The plan of this journal is comprised in the following passage; "the work will assume less the form of a review or digest of general improvements in medicine, than of a receptacle of the local results of original investigation. It will consist chiefly of papers, founded on clinical observations, or the results of pathological and physiological inquiry:—to these will succeed select cases, &c." We wish the journal every success, and we have no doubt that it will grow more practical as it gets older.

The first paper which we shall notice is the eighth in the order of succession —on the grinders' asthma. This is a term given to the disease by the patients themselves, and is in fact, that form of consumption which prevails amongst the workmen who are employed in grinding the different kinds of cutlery in Sheffield and its vicinity. It bears little or no resemblance, however, to asthma, and is probably so designated by the sufferers, from a natural desire on their parts, to conceal the fatal character of the disease.

"The articles which are ground in this neighbourhood are forks, awl-blades, fire-irons, razors, scissors, pen-knives, table-knives, large pocket-knives, files, joiners' tools, saws, sickles, and scythes. Some of these are ground on dry grind-stones, others on wet grind-stones, hence the grinders are divided into two classes, the dry, and the wet grinders — and there is a third class, who grind both wet and dry —altogether they amount to about two thousand five hundred, of this number about one hundred and fifty, viz. eighty men and seventy boys are fork grinders —these grind dry, and die from twenty-eight to thirty-two years of age.* The razor grinders grind both wet and dry, and they die from forty to forty-five years of age. The table-knife grinders work on wet stones, and they live to betwixt forty and fifty years of age. It may appear extraordinary that a disease, committing such ravages amongst so large a number of workmen, should have hitherto excited so little attention either in the profession or out of it. In most works which treat of pulmonary diseases, it is indeed mentioned that the fine particles of dust and steel, which are evolved in certain mechanical processes are frequent causes of consumption, but I am not aware that any treatise has been expressly written upon the subject, except by Hecquet in his 'Maladies des Artisans,' and Dr. Johnstone of Worcester, in the 5th vol. of the 'Memoirs of the Medical Society of London.' The former of these papers I have not seen, and the latter refers exclusively to needle pointing, a branch of grinding which is not carried on in this immediate neighbourhood. It must however be observed, that the grinders' asthma, as it exists amongst the Sheffield grinders, is a disease of recent origin. Until the beginning of the last century, grinding was not a distinct branch of business, but was performed by men who were also employed in forging, and hafting; hence they were exposed but seldom, and then only for a short time, to the pernicious effects of grinding. They worked also in large lofty rooms, which did not contain more than six or eight stones—were open to the roof—without windows—and with the cog-wheel always in the inside—thus, such a circulation of air was constantly kept up, that the small quantity of dust raised from these few stones was soon carried away. The wheels were always situated in the country—by the side of running streams, and frequently two or three miles from the habitations of the workmen, so that they had the advantage of pure air, and moderate exercise, in passing to and from their employment. Moreover, for several months during each Summer, they could not work more than four or five hours a day, owing to the scarcity of water. The grinders at that time lived chiefly in the country—had less intercourse with each other, and were consequently less exposed to those excesses which frequentlyprevail, where large bodies of workmen are congregated together—they were indeed distinguished for their simple manners, and temperate habits. This was the golden age of the grinders.

About the beginning of the last century the division of labour was gradually introduced into the manufacture of cutlery, and grinding became the sole

* Some exceptions to these general remarks may be met with, amongst those who have continued to work at open wheels in the country, and amongst others, who have been absent from their employment for many years together as soldiers.

employment of the grinder. Sometime after the middle of the same century, several grinders were observed to die of complaints nearly similar. The attention of their companions was excited, and they found the complaint was peculiar to themselves; still, however, it was far from being common, for they continued to enjoy all the advantages which their predecessors had possessed, except that being no longer employed in hafting and forging, they passed all their working hours at the grinding wheel.

Towards the close of the last century, it was found that the business of grinding had so much increased, that the grinding wheels already established were insufficient; but as every fall of water within five or six miles of Sheffield was occupied by wheels, it was impossible to add to their number. In this emergency, those connected with the trade resolved to avail themselves of the power of steam, and in the year 1786, the steam engine was applied to the purposes of grinding. A great revolution then took place in the circumstances of the grinder. He now worked in a small low room, where there were eight or ten stones, and sometimes as many as sixteen persons employed at one time. The doors and windows were kept almost constantly closed, a great quantity of dust was evolved from so many stones, and there was scarcely any circulation of air to carry it away. The steam engine, unlike the stream that had formerly supplied his wheel, allowed him no season of relaxation—it worked on an average eleven hours in the day, and six days in the week. The grinders began to reside more generally in the town—most of them lived near their respective wheels; their habits became less temperate, whilst the steady and industrious having now an opportunity of working as much as they pleased, died at an earlier age than even the idle and dissipated. So general has this destructive malady become of late years, that the result of some inquiries, made in 1822, shewed that out of two thousand five hundred grinders, there were not thirty-five who had arrived at the age of fifty, and perhaps not double that number, who had reached the age of forty-five: and out of more than eighty fork grinders, exclusive of boys, it was reported that there was not a single individual thirty-six years old!" *

This is a melancholy picture of the "march of intellect!" It is natural to suppose that humanity would endeavour to find out some remedy for the evil; and, accordingly many expedients were suggested. Dr. Johnstone proposed that the mouth and nostrils should be covered with crape; but this was soon found to be ineffectual, or rather impracticable, for the dust from the stone, and the moisture from the breath rendered the crape nearly impermeable, and then the heat and oppression of breath became intolerable. Mr. Abraham next suggested magnets so arranged as to intercept the particles of dust in their passage to the lungs. This invention was so highly valued that the Society of Arts rewarded the inventor with a large gold medal, and his fellow townsmen presented him with a service of plate, value one hundred pounds.

"Such were the favourable auspices under which this invention was submitted to the attention of the grinders; yet this 'life preserving apparatus' was never generally adopted by them, nor even partially for longer than five or six months. The trouble of arranging the magnets, and of removing the dust as it collected upon them, was too great for the grinders; besides, it was the metallic particles which the magnets were chiefly calculated to arrest, and there is reason to believe, from facts that will be adduced hereafter, that the grit dust is not only the most copious, but also the most injurious part of what is inhaled by the grinder. Mr. Abraham's merit, however, was not confined to the application of magnets for the relief of the grinders, he suggested another contrivance, which though less scientific has proved of more practical

* I am informed there is a fork-grinder above fifty years old who has worked at a steam-wheel—he is a man of irregular habits.

utility, by giving rise to that series of improvements, which have been since more or less adopted. It consisted of an additional apparatus which was formed of a piece of coarse sacking, or flannel, attached to a frame of wood; this was to be placed before the stone, and closely behind the safety guard of magnets, so as to secure all the dust which they had failed to arrest. This sacking or flannel was to be kept constantly wet, and the dust was to be shaken out of it when sufficient had been accumulated."

But this also failed, and all apparatus is now almost entirely abandoned. It is remarked by Dr. Knight that immediately after the application of steam to the purposes of grinding, this fatal malady increased rapidly—and that it is, at present, almost universal among those employed at grinding wheels worked by steam engines.

Dr. Knight next enters into a somewhat prolix notice of the opinions entertained by different writers respecting the influence of dust when inhaled into the lungs, as exciting asthma. These notices we shall pass over. The following is the rise and progress of this afflicting malady.

"Those who are to be brought up grinders, usually begin to work when they are about fourteen years old: there are, however, many exceptions to this custom, as the children of grinders are frequently employed in the lighter branches of the trade as early as eight or nine years of age. Such as are predisposed to pulmonary complaints soon begin to experience the injurious effects of grinding; and as at that time of life they are not too old to be put to other trades, they occasionally leave the wheel, and thus preserve both their lives and their health, whilst their more robust companions are sacrificing both. Grinders, who have good constitutions, seldom experience much inconvenience from their trade until they arrive at about twenty years of age: about that time the symptoms of their peculiar complaint begin to steal upon them, their breathing becomes more than usually embarrassed on slight exertions, particularly on going up stairs, or ascending a hill; their shoulders are elevated in order to relieve their constant and encreasing dyspnœa; they stoop forward, and appear to breathe the most comfortably in that posture in which they are accustomed to sit at their work, viz. with their elbows resting on their knees. Their complexions assume a dirty, muddy appearance; their countenance indicates anxiety; they complain of a sense of tightness across the chest; their voice is rough, and hoarse; their cough loud, and as if the air were driven through wooden tubes: they occasionally expectorate considerable quantities of dust, sometimes mixed up with mucus, at other times in globular or cylindrical masses enveloped in a thin film of mucus. Hæmoptysis frequently occurs; the blood is seldom florid or in large quantities; there is frequently a perceptible thickening about the larynx or trachea, with tenderness and cough on pressure. The expectoration is variable, frequently in small quantities, and frothy; in the more advanced stages, copious, and purulent; it is sometimes of a dusky red colour, and occasionally extremely fetid. The pulse, in the earlier stages, appears sometimes not to correspond with the severity of the disease, as it then frequently ranges from 80 to 90, when the concourse of other symptoms would lead us to expect that it should beat from 100 to 120: in the advanced stages, as in other cases of pulmonary consumption, it is almost uniformly quick. About thirty years of age the dry grinders become incapable of performing their usual labour, the wet grinders are similarly affected about forty. The sense of *fastness* under the sternum now grows very distressing; the lungs feel as if they were choked up with dust; the cough becomes incessant: the expectoration copious, and purulent; and the pulse quick; dropsy in some form or other not unfrequently supervenes. Hæmoptysis, inability to lie down, night sweats, colliquative diarrhœa, extreme emaciation, together with all the usual symptoms of pulmonary consumption at length carry them off; but not until they have lingered through months, and even years of suffering, incapable of working so as to support either themselves or their families."

The course of the disease, however, is frequently modified by the supervention of inflammatory attacks, rendering the original malady much more dangerous.

The treatment of Grinder's asthma is, unfortunately, very unsatisfactory. There can be little hope of cure, so long as the causes continue to be applied. The men seldom have recourse to advice till they are unable to work—and they return to labour as soon as they gain a little relief. Thus, he can never, with strict propriety, be said to be cured.

The treatment of the first stage is simple and obvious. Rest, an emetic, leeches, the antiphlogistic regimen, diaphoretics, mercurials, and saline aperients, constitute the whole of the treatment. Under these remedies the patient usually returns to work in ten days or a fortnight. In the second stage of the disease, the attack is more obstinate, and the relief less decided. Besides leeching and cupping, it is necessary to keep up counter-irritation on the external fauces and chest, by blisters, antimonial ointment, and setons. Digitalis and colchicum are useful; and mercury pushed so as slightly to affect the gums is sometimes beneficial. Even in this stage, recovery sometimes takes place, when the patients are removed from the operation of the causes. During the late war, grinders frequently went into the public service, and there regained their health. In the third or ulcerated stage, the case is hopeless, and medicines can only smooth the way to the grave. The pathology of the disease must be inferred from the symptoms, for it appears that hitherto no post-mortem examinations have been permitted—or at least made. We wonder much at this, for even in the most obscure villages, dissections are occasionally allowed by friends, when proper representations are made by the medical attendants.

---

